# Triazine-Mediated Zero-Length Crosslinking for Sustainable Leather Tanning

**DOI:** 10.3390/polym18161968

**Published:** 2026-08-12

**Authors:** Valentina Beghetto, Eleonora Fabris, Francesco de Laurentiis, Marco Nogarole, Domenico Santandrea, Dior Tall

**Affiliations:** 1 Department of Molecular Sciences and Nanosystems, University Ca’ Foscari of Venice, Via Torino 155, 30172 Mestre, Italy; eleonora.fabris.ef@gmail.com (E.F.); domenico.santandrea@unive.it (D.S.); dior.tall@unive.it (D.T.); 2 Crossing S.r.l., Viale della Repubblica 193/b, 31100 Treviso, Italy; 3 Consorzio Interuniversitario per le Reattività Chimiche e La Catalisi (CIRCC), Via C. Ulpiani 27, 70126 Bari, Italy; 4 Stazione Sperimentale Per L’industria Delle Pelli E Delle Materie Concianti S.r.l., Via del Lavoro, 22, 36071 Arzignano, Italy; f.delaurentiis@ssip.it (F.d.L.); m.nogarole@ssip.it (M.N.)

**Keywords:** zero-length crosslinking agent, green chemistry, triazine-based reagents, wet-white leather, collagen crosslinking, sustainable leather processing

## Abstract

The study presents a sustainable, metal-free tanning system based on 2-chloro-4,6-dimethoxy-1,3,5-triazine (CDMT) and *N*-methylmorpholine (NMM), which stabilizes collagen through a zero-length crosslinking mechanism. Reactive triazine intermediates, generated in situ, selectively activate collagen carboxyl groups, forming active esters that subsequently react with amine functionalities to form amide bonds. Unlike conventional tanning systems, no metals or toxic chemicals are incorporated into the tanned leather. Optimization of reagent concentration, temperature, and dosing strategy revealed that the gradual formation of reactive intermediates is essential to balance reaction kinetics and diffusion throughout collagen. Under pickle-free conditions, hydrothermal stability was achieved with only 2.5–3.4 wt% CDMT/NMM, yielding shrinkage temperatures of 81–85 °C that surpass most reported chrome-free tanning systems. The resulting leather displayed a bright white appearance, excellent dyeability, and outstanding physical-mechanical performance, including superior tear resistance and competitive tensile strength. These properties are consistent with the formation of a homogeneous collagen network reinforced by direct covalent amide crosslinks while maintaining fiber flexibility. Furthermore, avoiding pickling significantly reduces chemical consumption and improves wastewater biodegradability, enhancing the environmental sustainability of the process. Overall, CDMT/NMM emerges as a scalable, environmentally friendly tanning technology that combines mechanistically controlled collagen crosslinking with excellent leather performance.

## 1. Introduction

Leather processing, the oldest example of the circular economy, transforms a putrescible byproduct of the meat industry into extraordinary products for many different fashionable applications [1,2]. Yet to date, the industry struggles to move away from conventional leather processing practices, thereby having a significant environmental and social impact. In fact, currently more than 85% of leather goods are manufactured using Cr(III) salts, producing what is known as “wet blue” leather. This predominance is attributed to the high quality of the resulting leather, as well as economic efficiency, adaptability, and consistency [2]. Despite its advantages, chrome tanning generates substantial amounts of chromium-containing solid sludge, contributing to high disposal costs and significant environmental impacts [3,4,5].

The remaining 15% of production relies on alternative tanning systems, including chrome-free inorganic salts (such as aluminum, titanium, or zirconium) or metal-free agents like aldehydes and natural or synthetic tannins, yielding chrome-free leather [3,6,7,8,9,10,11]. Vegetable tannins are viewed as promising, hazard-free biobased alternatives; however, their industrial application remains limited due to the higher cost, comparatively weaker physical-mechanical properties of vegetable-tanned leather, and chemical oxygen demand (COD) and biological oxygen demand (BOD) levels in wastewater [12,13,14,15]. Alternatively, vegetable tannins or glutaraldehyde are often combined with aluminum salts, enhancing hydrothermal stability and organoleptic characteristics, though at the expense of versatility and environmental sustainability [3,9].

**Figure 1:**
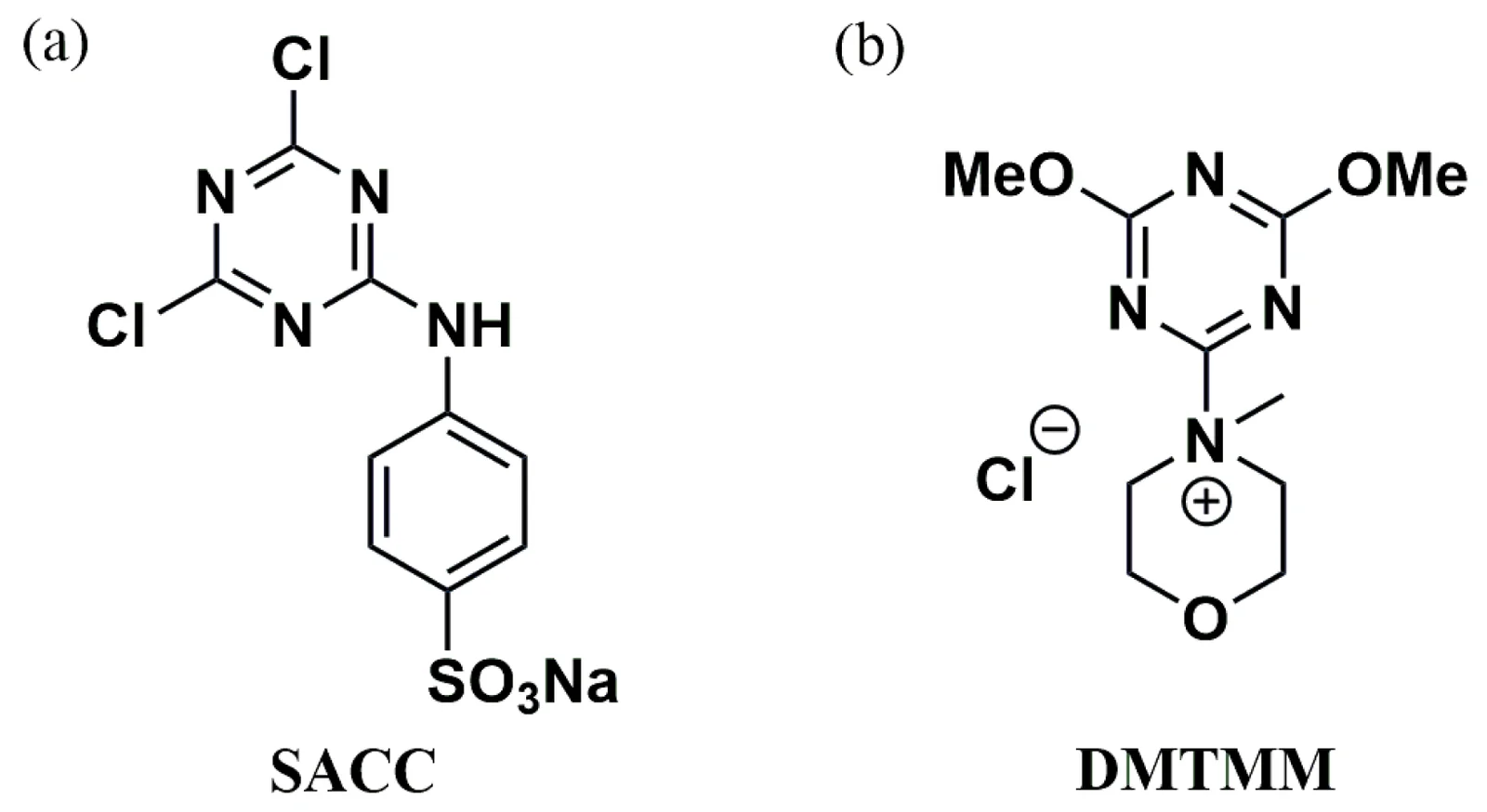
Chemical structures of representative triazine-based reagents used in leather tanning: (**a**) SACC; (**b**) DMTMM.

Recently, triazine-based tanning agents have emerged as sustainable alternatives for the production of chrome-free leather, avoiding the use of toxic organic compounds, generally employed as chrome alternatives, such as aldehydes, phenols, bisphenols, among others [16,17,18]. Sodium *p*-((4,6-dichloro-1,3,5-triazin-2-yl)amino)benzene sulphonate (SACC), sold as Granofin F90^®^ (Figure 1), is identified by ECHA as a substance of low concern, producing good quality leather with low environmental impact [19,20]. An interesting peculiarity of Granofin F90^®^, and in general of triazine-based tanning agents, is the possibility to avoid pickling before tannage, reducing the consumption of high quantities of NaCl, as well as strong and weak acids and bases, and consequently reducing the load of pollutants in wastewater [16]. Pickling contributes significantly to total dissolved solids (TDS) and chloride emissions in tannery wastewater, accounting for a major environmental burden in leather processing. The ability of triazine-based systems to operate without pickling, therefore, offers substantial environmental and economic advantages. Some different examples of triazine-based compounds can be found in the literature, which can be divided into SACC analogs, 2,4,6-trichloro-1,3-5-triazine-derived tanning agents, and triazine quaternary ammonium salts, for example, 4-(4,6-dimethoxy-1,3,5-triazin-2-yl)-4-methylmorpholinium chloride (DMTMM) (Figure 1) [17,19,20,21,22,23,24,25,26,27].

**Scheme 1:**
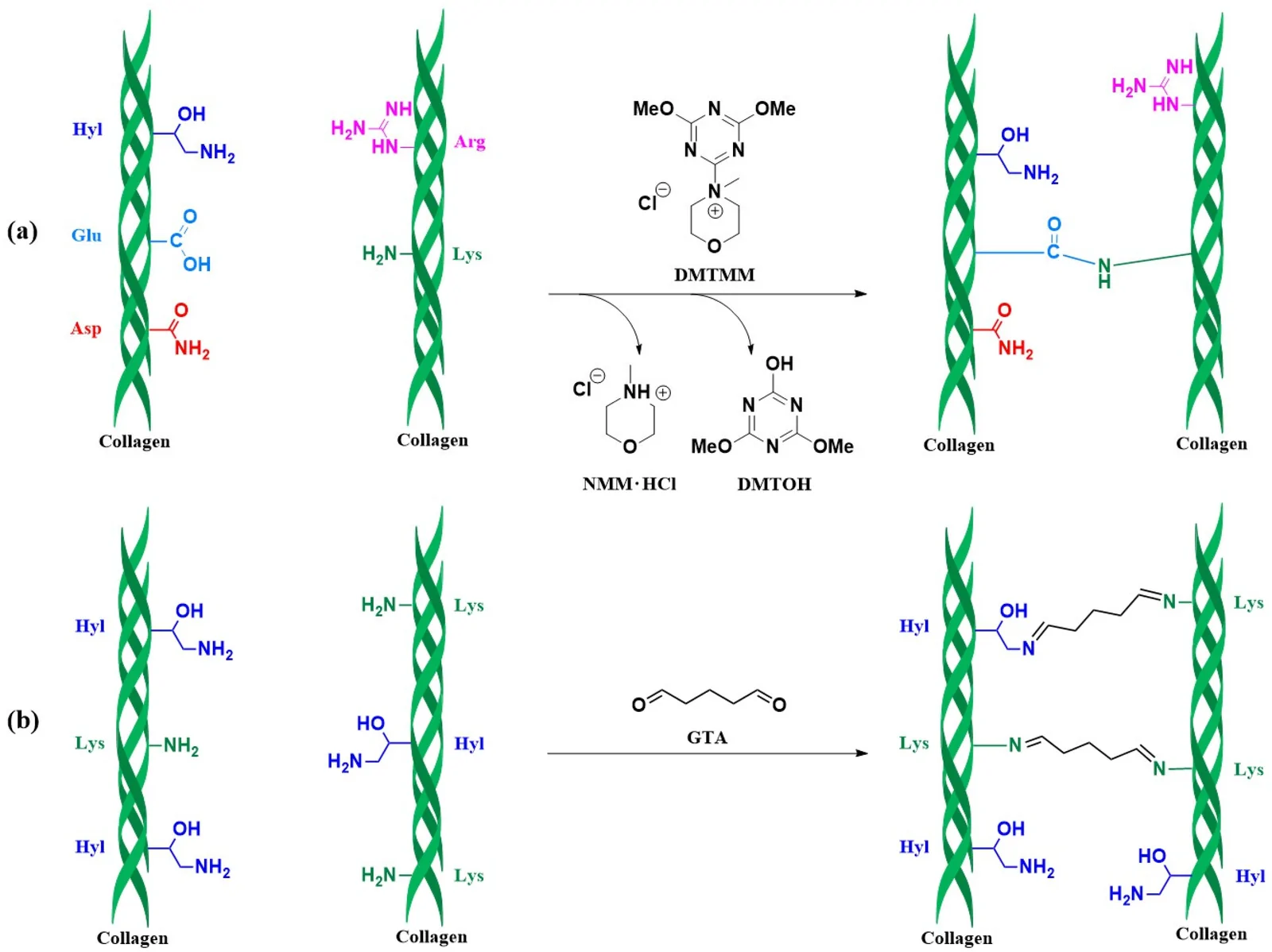
Comparison of collagen crosslinking mechanisms: (**a**) zero-length amide bond formation mediated by DMTMM; (**b**) conventional aldehyde-mediated crosslinking (glutaraldehyde).

DMTMM has been extensively investigated as a coupling agent in the presence of proteins [28,29,30,31,32,33,34,35], peptides [31,36,37,38,39,40,41,42,43,44], and other materials [45,46,47,48,49,50]. From a mechanistic perspective, the stabilization of collagen during tanning is governed by the formation of crosslinks between functional groups of amino acid side chains, primarily carboxyl (Asp, Glu) and amine (Lys, Hyl) residues. Conventional tanning systems rely either on coordination chemistry (chromium salts) or covalent modification via chemical agents (aldehydes, SACC). In contrast, DMTMM is a zero-length crosslinking agent that operates under a fundamentally different paradigm, promoting direct bond formation between intrinsic functional groups of collagen without introducing organic chemicals or metal complex linkers into the matrix [42,51,52,53]. The accepted reaction pathway [27,42] involves activation of a carboxylic group by the 1,3,5-triazine ring to generate an “active ester” intermediate, which subsequently reacts with an amine to form an amide linkage. During this process, DMTMM breaks down into 2,4-dimethoxy-6-hydroxy-1,3,5-triazine (DMTOH) and 4-methylmorpholinium chloride (NMM·HCl), both non-toxic, water-soluble byproducts (Scheme 1).

Notably, the chlorine atom in the triazine structure does not release HCl; instead, it reacts with the tertiary amine to generate the stable quaternary ammonium salt NMM·HCl. This feature minimizes corrosion and prevents collagen damage associated with the lyotropic effects of free HCl [2].

**Scheme 2:**
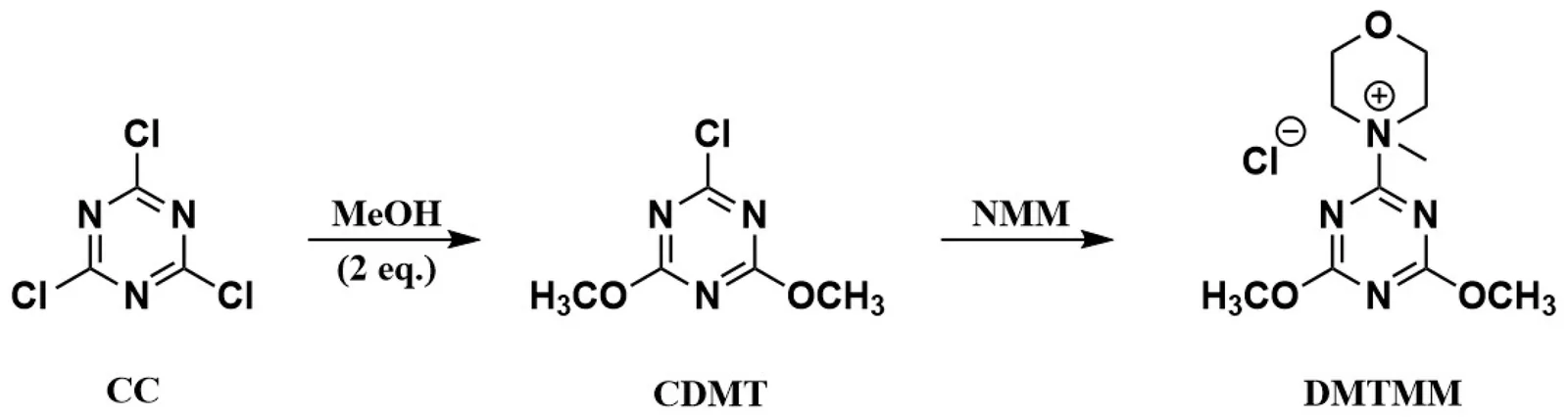
Synthetic pathway of CDMT and its conversion to DMTMM, highlighting the multistep nature of DMTMM preparation.

Presently, although promising from an environmental point of view, triazine-based tanning agents are still striving to reach wide industrial application due to low solubility and reactivity (SACC) or, for DMTMM, high production costs derived from its multistep synthesis (Scheme 2), together with its excessive reactivity towards leather, generating wrinkling problems [16,54].

Therefore, the aim of this work is to develop and evaluate a novel pickle-free, metal- and aldehyde-free tanning system based on the binary CDMT/NMM system as a zero-length crosslinking platform for collagen stabilization. The proposed approach seeks to overcome the limitations of DMTMM by enabling in situ generation of reactive triazine intermediates, thereby improving control over reactivity, reducing reagent consumption, and enhancing leather quality. The performance of the CDMT/NMM system was systematically investigated in terms of thermal stability, organoleptic properties, and mechanical behavior of tanned leather produced, and benchmarked against both DMTMM and glutaraldehyde (GTA) tanning systems. Particular attention is devoted to process efficiency, environmental impact, and applicability to industrial sectors such as automotive, upholstery, and footwear, with the objective of establishing CDMT/NMM as a viable and sustainable alternative to conventional tanning technologies.

## 2. Materials and Methods

### 2.1. Materials

Solvents and chemicals were purchased from Sigma Aldrich (Milan, Italy) and used without further purification. Bovine and bull hides used for the tanning tests were purchased from the Stazione sperimentale per l’industria delle pelli e delle materie concianti (SSIP) of Arzignano (Arzignano, VI, Italy).

### 2.2. Methods

The shrinking temperature (Ts) of DMTMM tanned leather was measured according to EN ISO 3380 (IULTCS/IUP 16, 2015) [55]. A leather sample was suspended vertically in water, and the rate of heating was maintained at 2.0 ± 0.2 °C/min. The Ts is the temperature at which the leather shrinks one-third of its original length. The physical and mechanical properties, such as tensile strength, tearing load, elongation at break, maximum force, distention and force at crack, color fastness, and hot shrinkage, were determined according to standard methods and the Fiat specifications of crust leathers [56,57,58,59,60,61]. The mechanical properties of CDMT/NMM leather were determined on eight parallel samples using a Dynamometer AG/MC1 (Acquati, Milan, Italy) for the determination of tensile strength and percentage elongation, by method UNI EN ISO 3376 [56] and for the determination of tear load, following the UNI EN ISO 3377-1 method [57]. Determination of distention, strength surface crack (ball burst method), and force at crack were measured with a Lastometer I.U.P./9 IG/LAST/D (Giuliani Technologies, Turin, Italy), by the UNI EN ISO 3379 method [58]. Rubbing fastness tester FEK-VESLIC (Giuliani Technologies, Turin, Italy) was used for color fastness to cycles of to-and-fro rubbing testing, by method UNI EN ISO 11640:2013 [59]. Concerning Fiat specification 9.55253, the dimensional stability of a disk (200 mm diameter) was tested by heating it in an oven at 110 ± 2 °C for 6 h, then the sample was allowed to cool to 23 ± 5 °C. The diameter of the sample was measured in two perpendicular directions, and the average radius and area were calculated. The limit for the Fiat specification is an area reduction of a maximum 3% [60]. Prior to performing physical tests, all samples were conditioned according to the standard EN ISO 2419 [61]. Organoleptic properties of crust leather samples were assessed for softness, fullness, grain flatness, grain smoothness, grain tightness, and general appearance by hand and visual examinations by experienced tanners (on a scale of 1 to 10). The pH, Chemical Oxygen Demand (COD), chlorine concentration, Total Kjeldahl Nitrogen (TKN), and Total Suspended Solids (TSS) of wastewater collected after tanning were measured according to APAT-IRSA standard methods [62] on wastewater collected after tanning tests. The synthesis of CDMT and Tanning trials are described below.

#### 2.2.1. Synthesis of 2-Chloro-4,6-Dimethoxy-1,3,5-Triazine (CDMT)

The synthesis of CDMT was carried out according to the procedure reported by Morandini et al. [47]. In a 3000 mL round-bottomed flask, equipped with a magnetic stirring bar, a solution of sodium hydrogen carbonate (260.0 g, 3.0 mol) in methanol (2000 mL) was prepared. After cooling at 0 °C, the cyanuric chloride (228.7 g, 1.24 mol) was added, and the resulting mixture was stirred at room temperature for 2.5 h, then at 55 °C for 2 h. The suspension was filtered, and the solid was washed with water and vacuum-dried (185.1 g, 1.05 mol) to give 85% yield. ^1^H NMR (300 MHz, CDCl_3_) δ (ppm): 4.08 (6H, s, OCH_3_). ^13^C NMR (75 MHz, CDCl_3_) δ (ppm): 172.6, 172.1, 55.4.

#### 2.2.2. Tanning Tests

Preliminary tanning tests were carried out on bathed bovine hides, 25 × 20 cm in size (approximately 0.15 to 0.2 kg), and then repeated on bull hides (7–10 kg) (Tables S1–S3). All tanning experiments were performed in triplicate. A bull hide weighing about 16 kg was cut in half and used for a comparison of CDMT/NMM and glutaraldehyde tanning.

**Tanning protocol with CDMT/NMM**: tests were carried out using the binary tanning system CDMT/NMM, according to a patented procedure [34,63]. In a drum containing delimed and bathed hides (50 wt/wt water), variable wt% of solid CDMT were added, while NMM was diluted in water (30 wt%, CDMT/NMM 1.5/1.0 wt/wt by weight of hide) and poured through the liquid dosage system of the tanning drum, in up to four doses as reported in Table 1 and in Supplementary Materials. The time between each addition was 2 h, and the temperature varied, depending on the tanning conditions tested, between 15.0 °C and 33.0 °C (Table 1). Once the additions were complete, the drum was kept rotating for a further 18 h (Tables S1 and S2). Tanning progress was monitored by assessing the shrinkage temperature (Ts) of each leather sample prior to the addition of each dose, according to the IULTCS/IUP 16 (EN ISO 3380:2015) method [55]. The tanned hides were dried, and their Ts values were further measured. Three post-tanning protocols were adopted to obtain crust leather (Tables S4 and S5).

**Tanning protocol with glutaraldehyde**: tests were carried out on bull hide with 2.5 wt% glutaraldehyde, 3.5 wt% of a synthetic tannin (4,4′-sulfonyldiphenol), in 50 wt% water by weight of the hide (see Table S3). After 18 h, the tanned hides were dried, and their Ts measured [55]. A conventional post-tanning process was adopted to obtain the crust leathers using sulphonated fish oil, sulphosuccinate, methacrylic resin, Tara powder, and acid brown 282 (see Table S6).

**Tanning protocol with DMTMM**: tests were carried out on a bovine hide with 2.5 wt% of DMTMM and 50 wt% water by weight of hide. After 18 h, the tanned hide was dried, and their Ts measured [55].

## 3. Results

### 3.1. Tanning with Zero-Length Crosslinking Agent

**Scheme 3:**
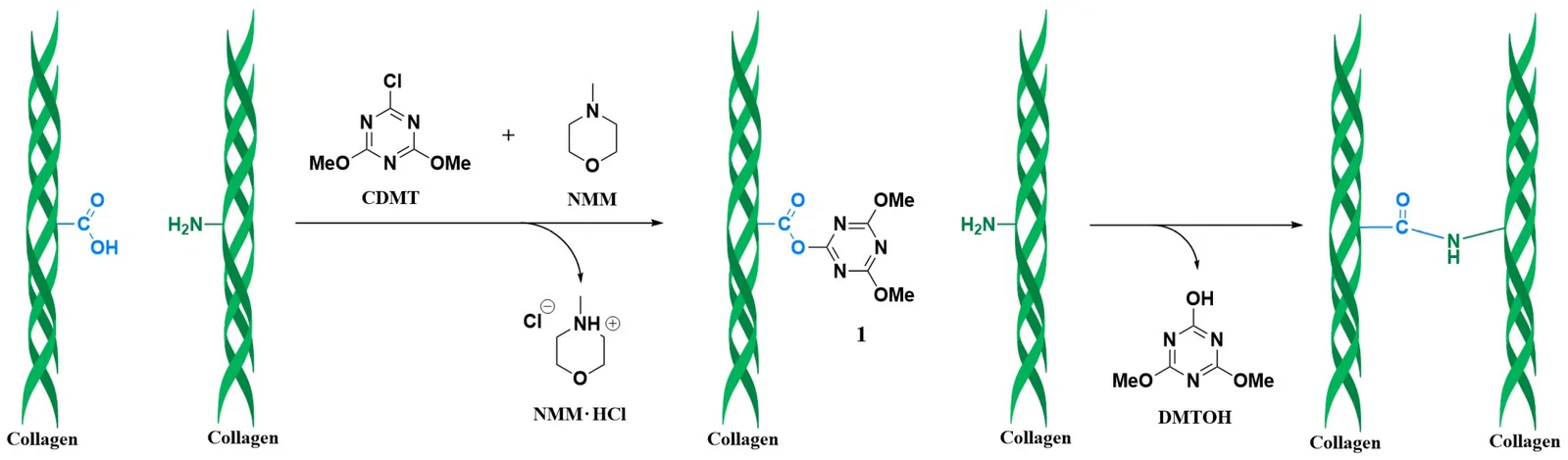
Possible pathway for collagen amidation in the presence of 2-chloro-4,6-dimethoxy-1,3,5-triazine (CDMT)/*N*-methyl morpholine (NMM) system.

The tanning activity of the CDMT/NMM system can be rationalized based on established triazine-mediated condensation chemistry [64]. In aqueous conditions, CDMT reacts with NMM to generate reactive species capable of activating the carboxylic groups of collagen, forming an active ester that subsequently reacts with nucleophilic amine groups to yield stable amide bonds. This process constitutes a zero-length crosslinking mechanism, as covalent bonds are formed directly between intrinsic functional groups of collagen without the tanning agent being incorporated into the final matrix (Scheme 3). Additionally, NMM acts as an HCl scavenger, preventing local acidification and preserving collagen integrity [42,64]. A key distinction from DMTMM lies in the in situ generation of reactive species. While DMTMM is a pre-formed, highly reactive coupling agent, the CDMT/NMM system produces reactive intermediates progressively, allowing better control over reaction kinetics [47]. As reported below, this difference is critical in modulating the balance between surface reactivity and bulk diffusion within the collagen network. The gradual decrease in pH observed during tanning (from ca. 7.8 to ~5.5–5.8, Table 1) is consistent with the formation of an equimolar amount of acidic by-products such as 2,4-dimethoxy-6-hydroxy-1,3,5-triazine (DMTOH), in agreement with the literature [27]. This pH change provides an indirect and useful measure of reagent consumption and reaction progress.

It is important to note that experiments confirm that the binary CDMT/NMM tanning system does not require pickling prior to tanning, thereby reducing the environmental impact of the process (see below). Additionally, the absence of pickling not only reduces environmental impact but also alters collagen fiber accessibility. Under near-neutral conditions, electrostatic repulsion between collagen chains is minimized compared to acidic pickling conditions, potentially facilitating more uniform penetration of the tanning agents. This may contribute to the improved homogeneity and reduced surface defects observed in CDMT/NMM-treated leathers.

Initial experiments (T0) were carried out on a bovine hide (~0.15 kg), using 8.4 wt% of the tanning agent (CDMT/NMM 7.6/5.0 g/g) relative to the hide weight in 50 wt% water. Solid CDMT was added together with the hide in the drum, while NMM (30 wt% solution) was added through the liquid dosage system of the drum.

**Figure 2:**
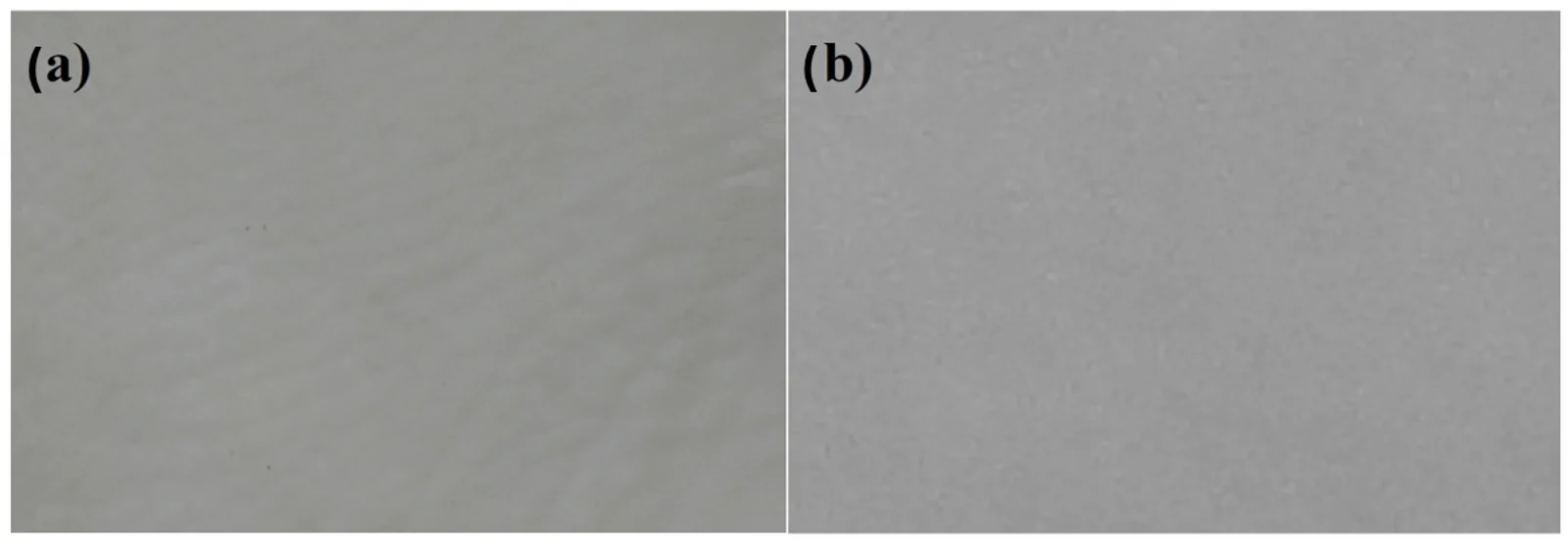
Visual comparison of tanned leather hides. (**a**) Bovine hide tanned with 8.4 wt% CDMT/NMM, single dosage (T0), and (**b**) four rates (T1), showing different surface texture properties.

**Figure 3:**
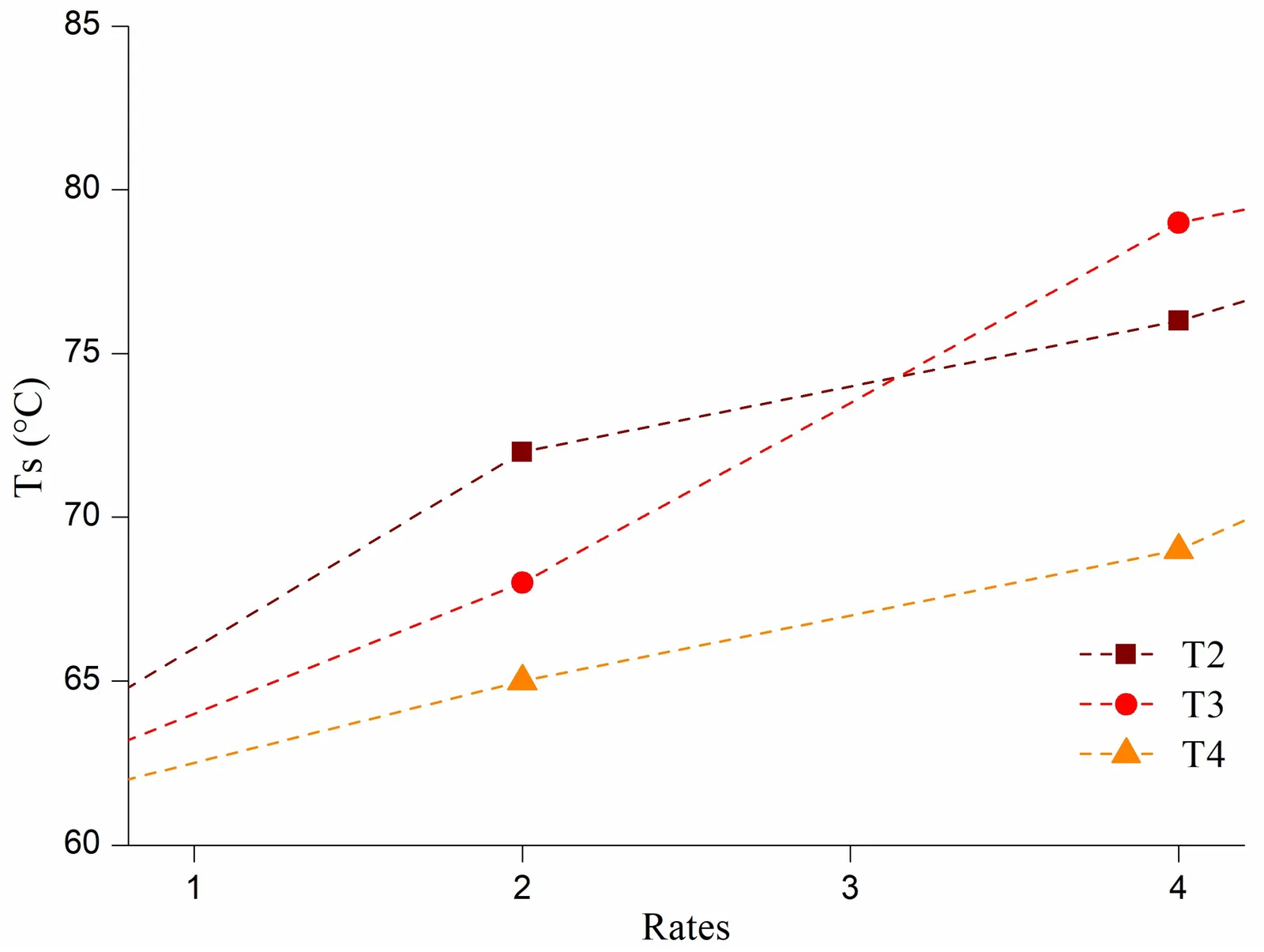
Shrinkage temperature (Ts) of leather samples as a function of application rates during tanning experiments (T2–T4) carried out at 15.0 °C (T2), 25.2 °C (T3), and 33.7 °C (T4).

However, the addition of CDMT/NMM at a single dosage led to poor organoleptic properties, characterized by pronounced surface wrinkling (Figure 2a). This behavior can be attributed to a kinetic imbalance between reaction kinetics and diffusion: rapid crosslinking at the grain surface limits the penetration of reactive species into the inner fiber structure, generating surface wrinkling phenomena. To address this limitation, a stepwise addition protocol was implemented. Distributing the total reagent load over multiple additions (every 2 h) significantly improved leather quality while maintaining comparable Ts values (see T1 in Table 1 and Figure 2b). Although diffusion was not measured directly, the progressive increase in shrinkage temperature during sequential addition (Figure 3), together with the improved grain morphology observed between T0 and T1 (Figure 2), strongly supports this interpretation.

Importantly, this behavior contrasts with DMTMM, where excessive intrinsic reactivity often leads to similar surface defects even under controlled conditions. The CDMT/NMM system, therefore, offers a practical advantage by enabling reactivity tuning through process parameter control rather than relying solely on chemical structure. Aiming to gain further evidence on the proposed cross-linking mechanism, ATR FT-IR analysis of untanned hide and T0 were compared (Figure S1), yet, due to the intrinsic abundance of amide functionalities in the collagenous matrix, the contribution of additional amide bonds was not evidenced. Thus, shrinkage temperature (Ts) was employed as an indirect yet highly effective diagnostic tool to evaluate crosslinking efficiency. SEM analysis further confirms the formation of a more compact and ordered collagen fiber arrangement compared to the loosely structured bundles in the untanned hide (Figures S2 and S3).

Systematic reduction in the total reagent loading from 8.4 wt% to 2.5 wt% demonstrated that high shrinkage temperatures (>80 °C) can be maintained even at low concentrations (Table 1). Compared to other triazine-based systems reported in the literature, this performance is particularly significant (Table S7). For example, SACC typically requires higher loadings (≥4 wt%) and, in some cases, elevated temperatures or a combination with auxiliary agents to reach similar Ts values [1,19,20,21,22]. In contrast, the CDMT/NMM system achieves comparable or superior thermal stability under milder conditions and without increasing formulation complexity.

Temperature also plays a critical role in system performance. Optimal results were obtained at moderate temperatures (~20–25 °C), while higher temperatures led to reduced efficiency (Figure 3). In particular, different experiments were performed at temperatures between 15.0 and 33.2 °C, and the temperature of the hides (Ts) was monitored every two hours before the 2nd, 3rd, and 4th addition of CDMT/NMM, and after 18 h (Table 1 and Figure 3). Data reported in Figure 3 indicate that a higher tanning efficiency was registered when the initial water temperature (Tin) was 25.2 °C, although the same final Ts was also measured when the Tin of the tanning bath was 15.0 °C. On the contrary, if Tin is above 33.0 °C, a moderately lower efficiency of the tanning agent was observed, reaching Ts of 78 °C by 18 h. This behavior is consistent with the known instability of triazine-based systems at elevated temperatures, which can result in premature deactivation [31]. These findings further support the importance of balancing reactivity and stability to maximize crosslinking efficiency. It should nevertheless be considered that T > 30.0 °C is generally avoided, also to prevent degradation of native collagen, which starts at T > 40–45 °C. Additionally, data reported in Table 1 further evidence that no significant variation of Ts was observed after the third addition of CDMT/NMM (6 h). So, further experiments were carried out with lower tanning agent concentrations (down to 2.5 wt%), added in three doses, reducing CDMT/NMM consumption and tannage duration. In fact, in these tanning conditions (tests T5–T8, Table 1), no relevant difference in Ts was observed either for the reduction in reagents wt% load nor for the addition mode (three instead of four doses). Nonetheless, if quantities below 2.5 wt% of the binary system were used by weight of hide, the Ts decreased significantly (Ts 73 °C with 2.0 wt%); therefore, 2.5 wt% of CDMT/NMM was selected for further studies. Interestingly, Ts obtained with 2.5 wt% of CDMT/NMM (Ts 81.0 °C) are among the highest reported in the literature for triazine or, in general, for metal-free tanning agents, employing such very small amounts of tanning agent [1,19,20,21].

For example, when 4 wt% of SACC (based on leather weight) is used, the resulting Ts reaches approximately 78 °C, comparable to the least performing conditions for CDMT/NMM (Table 1), and slightly exceeding the minimum threshold required for subsequent splitting and shaving operations (Ts > 75 °C). Moreover, unlike combination systems employing triazines and vegetable tannins or auxiliary agents, CDMT/NMM achieves high performance without increasing reagent consumption or environmental burden [20]. It should be noted that although normal tanning practices involve leaving hides to react overnight, CDMT/NMM efficiently crosslinks collagen by 6 h, potentially reducing processing times and improving plant productivity.

A direct comparison between CDMT/NMM, DMTMM, and GTA systems provides valuable insight into the advantages of the proposed approach. At equivalent reagent loadings (2.5 wt%), CDMT/NMM achieves shrinkage comparable to GTA and significantly higher than that obtained with DMTMM (T9–T10, Table 1). The lower performance of DMTMM under identical conditions can be attributed to its excessive reactivity, which promotes rapid surface crosslinking and limits effective penetration. In contrast, the CDMT/NMM system benefits from controlled generation of reactive intermediates, enabling deeper diffusion and more uniform stabilization.

From an environmental perspective, the comparison is equally relevant. While GTA represents a widely used chrome-free alternative, it introduces aldehyde functionalities that are associated with toxicity and regulatory concerns. In contrast, CDMT/NMM combines the absence of metals with the elimination of aldehydes, providing a safer and more sustainable solution (see below).

**Figure 4:**
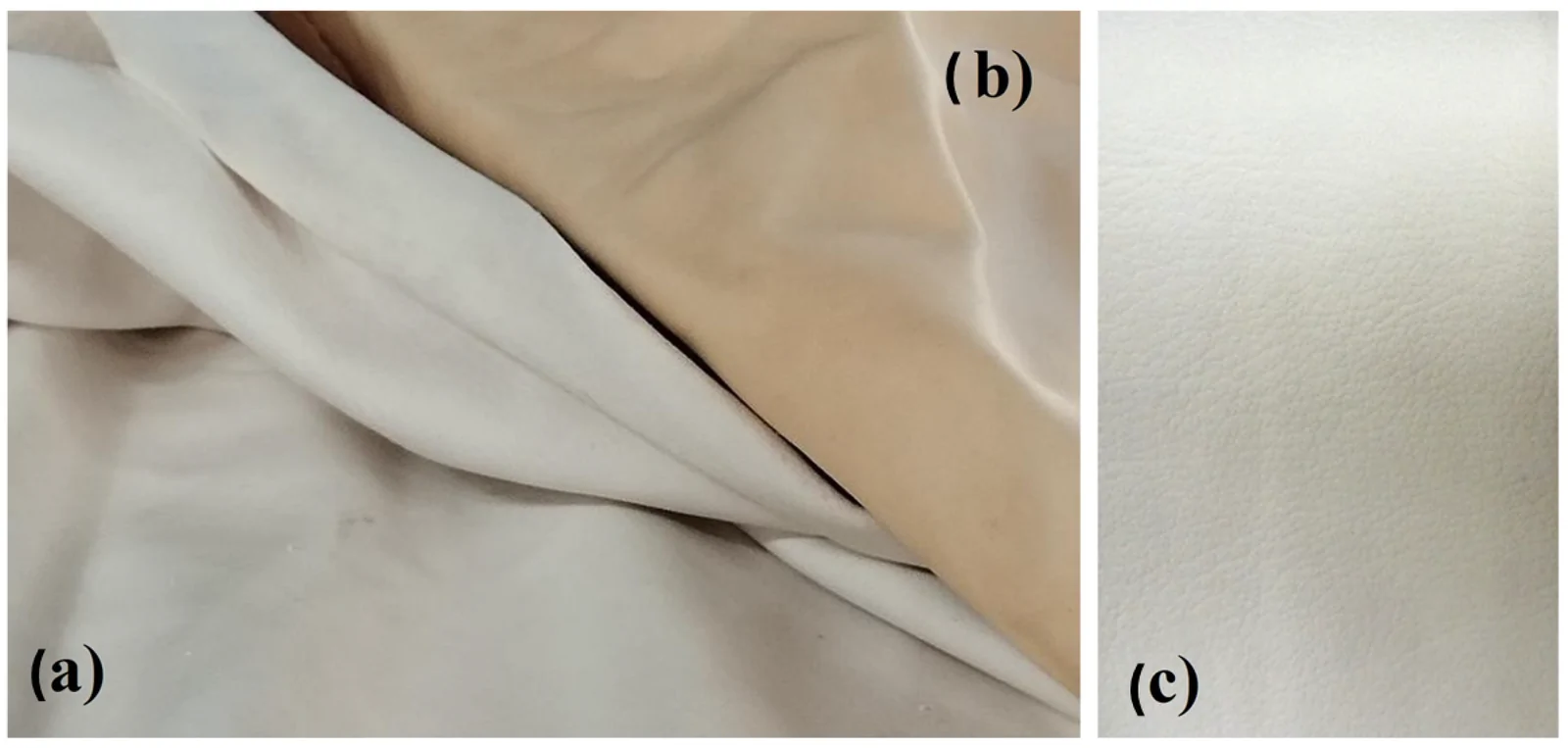
Comparison between (**a**) CDMT/NMM, (**b**) GTA, and (**c**) DMTMM tanned leather.

A further distinguishing feature is the color of the resulting leather. CDMT/NMM-treated samples, like DMTMM, exhibit a bright white appearance, whereas GTA-tanned leather typically shows a yellowish tone (Figure 4).

This difference is highly advantageous for downstream processing, as it enables greater flexibility in dyeing and allows the production of brighter and more uniform colors. Based on the results above, DMTMM was not used in subsequent comparison studies for the retanning, dyeing, and fatliquoring tests.

### 3.2. Physical-Mechanical Properties of Retanned Leather

Different retanning, dyeing, and fatliquoring formulations (R1, R2, R3; for details, see Tables S4–S6) were tested on hides tanned according to protocols T3 and T8, to prepare leather for upper footwear, automotive, and upholstery applications. Two protocols (R1 and R2; see Tables S4 and S5) were specifically formulated to verify the final characteristics of crust leather tanned with the innovative CMNT/NMM binary system for footwear (R1), automotive and upholstery (R2), while the third retanning protocol (R3), a standard procedure for the production of upholstery and automotive applications, was used to compare physical mechanical properties of CDMT/NMM and GTA leather.

At the end of the retanning process, the leather samples were air-dried at 25 °C for 18 h, then vacuum-dried at 35 °C for 5 min and tumbled and padded to soften them prior to characterization (Table 2).

Tensile strength (TS, MPa), Elongation at break (E%), Tear load (TL, N), Maximum tensile force at break (MF, N) were measured parallel or perpendicularly to the hide backbone (see Figures S4–S7; Supplementary Materials), while average values are reported in Table 2, together with standard requirements for upper upholstery, upper footwear and automotive crust leather according to the standards generally adopted as reference by SSIP. Further ball burst tests, color fastness, Fiat specification requirements, and organoleptic properties were carried out as reported in Section 2.

As evidenced by the data reported in Table 2, TS values of all crust leather samples tanned with CDMT/NMM or glutaraldehyde satisfy requirements for upholstery (TS ≥ 8 MPa), while only T3-R1 had adequate TS for more stringent upper footwear applications (>15.0 MPa), with values significantly higher than GTA (TS 15.9 MPa compared to 12.3 MPa of T9-R3, Table 2).

As far as E% is concerned, T3-R2 and crust GTA leather (T9-R3) are within the requirements for upholstery (E% between 35 and 75%), while other samples have E% which are higher than acceptable values for the different applications tested. Tear load (TL) measurements evidence that all CDMT/NMM crust leather samples have high TL suitable for upholstery and automotive applications, while only T3-R2 exceeds requirements for upper footwear (TL ≥ 100 N), reaching a very high value of 135.3 N, 140% superior to that of GTA crust leather (97.2 N, Table 2).

Another important parameter measured was the maximum tensile force at break (MF), which indicates the highest load the leather sample can withstand before it fails, reflecting its ultimate strength. It provides insight into the leather’s durability and resistance to tearing under tension, which is critical for assessing performance in practical applications like footwear or upholstery. As far as CDMT/NMM crusts are concerned, a substantial increase in MF for T3-R1 and T3-R2 was observed compared to GTA (256.3 N, 208.8 N, 163.4 N, respectively; Table 2), thus satisfying the requirements for all applications tested.

Distension and strength surface at crack (DC, ball burst method) and force at crack (FC) were also carried out according to ISO 3379:2015 [58]. This method is applicable to all flexible leathers and is particularly suitable for determining the durability of leathers for upper footwear. DC and FC values obtained for all samples are within acceptable limits for all applications tested, and an outstanding force at crack of 446.0 N was measured for T3-R1 (see Table 2 and Figure S8). Values obtained from the color fastness to cycles of to-and-fro rubbing test show adequate performances in line with ISO 3380:2015 for upholstery and upper footwear (Figure S8), while higher resistance necessary for the automotive was not obtained, also for T9-R3, probably indicating that further tuning of the retanning or dying process would be beneficial.

Regarding the hot-shrinkage Fiat specification requirements, leather disks measuring 200 mm were used, resulting in an area of 10,000 π mm^2^. The disk for T8-R3, at the end of the test, had an average radius of 98.5 mm, resulting in an area of 9702.25 π mm^2^ and a difference from the initial value of area of 3.0%, as reported in Table 2, within the limit imposed by Fiat for automotive leather interiors [60]. This does not apply to GTA crust leather (T9-R3), as at the end of the test, the average disk radius was 97.5 mm, resulting in an area of 9506.25 π mm^2^ and a difference from the initial value of 5.0%, thus exceeding the limit of 3.0%.

In addition to mechanical performance, organoleptic properties, including softness, fullness, grain uniformity, surface smoothness, and overall visual appearance, were assessed through tactile and visual evaluation to determine the quality of the tanned leather. The CDMT/NMM-treated samples exhibited characteristics comparable to those obtained with GTA tanning, with overall ratings in the range of 7–9. Physical-mechanical data for retanned crust demonstrate that the CDMT/NMM system provides leather with suitable properties for a wide range of industrial applications. Tensile strength, elongation at break, and tear resistance values meet or exceed standard requirements for upholstery, automotive, and, in selected cases, footwear applications. The observed mechanical performance is consistent with the proposed zero-length crosslinking mechanism, leading to the formation of a stable and well-connected network capable of distributing mechanical stress effectively.

At the same time, the absence of bulky or rigid organic compounds preserves the intrinsic flexibility of the collagen structure, contributing to favorable elongation properties. Notably, the high tear resistance observed in several formulations suggests that the CDMT/NMM system promotes uniform fiber cohesion without excessive stiffening, a balance that is often difficult to achieve with alternative tanning agents.

### 3.3. Environmental Impact of CDMT/NMM and Conventional Tanning Agents

Leather processing is widely recognized as a resource-intensive industry, characterized by high consumption of water and chemicals, and the generation of significant volumes of polluted effluents containing salts, organic matter, and hazardous substances. A major environmental advantage which CDMT/NMM system shares with DMTMM and SACC lies in the elimination of the pickling and basification steps [16,65]. Conventional tanning processes require acidic conditions (pH 2–5) in the presence of large amounts of sodium chloride (typically 8–10 wt%) to facilitate the penetration of tanning agents into the collagen matrix. This step contributes substantially to total dissolved solids (TDS) and chloride loads in wastewater, which are difficult to remove and represent a major environmental burden.

In contrast, systems operating under near-neutral conditions, avoiding the use of salts, strong acids, and alkali, and reducing chemical use (~100 kg/ton of hide) and water consumption (1000 kg/ton of hide) [4]. This simplification not only lowers the environmental footprint but also reduces process complexity and operational costs. To further understand the different environmental impact of CDMT/NMM, wastewater collected after tanning was analyzed to determine chemical oxygen demand (COD), biological oxygen demand at 5 days (BOD_5_), biodegradability of waste streams (BOD_5_/COD), total solids content (TSC), total dissolved solids (TDS), total suspended solids (TSS), total dissolved chlorines (TDC) and concentration of Cr(III) species. Data obtained were compared with those available in the literature for CTP, GTA, and triazine-based systems (Table 3).

CDMT/NMM avoids sodium chloride consumption (~80 kg/ton of hides processed), and, consequently, total dissolved chlorines present in wastewater (TDC) by over 90%, decreases total suspended solids (TSS) value by 40–60%, TDS by 30–40%, and TSS by 40–60% compared to CTP. As far as glutaraldehyde is concerned, wastewater quality is similar to that of CDMT/NMM except for two significant features, which are the absence of hazardous glutaraldehyde in waste streams and the biodegradability of wastewater. This parameter is measured by the ratio between the biological oxygen demand at five days and chemical oxygen demand (BOD_5_/COD) [66]. According to the literature, BOD_5_/COD of CTP, GTA, and most triazines is <0.25 (difficult to biodegrade), while for CDMT/NMM, this value is significantly above >0.45, considered the threshold value for easily biodegradable wastewaters [67]. Interestingly, values are even better than those measured for DMTMM, as a consequence of the low quantity of CDMT/NMM employed for tanning. From a sustainability perspective, the absence of residual tanning agents within the collagen matrix is particularly important. While further life cycle assessment (LCA) studies are required to fully quantify its environmental benefits at an industrial scale, current evidence strongly supports the potential of the CDMT/NMM tanning system as a low-impact, environmentally sustainable alternative to conventional tanning technologies.

Although a Life Cycle Costing analysis is beyond the scope of this work, considering the bill of materials, an average cost of ~3.79 €/kg was calculated for CDMT. Prudentially forecasting an incidence of production costs and distribution of 30% of the bill of materials, the final price of CDMT could be ~4.92/kg. Since to tan 1000 kg of hides, 50.7 kg of CDMT and 33.3 kg of NMM (~0.5 €/kg) are required, an average cost of €0.21 per kg of leather is estimated. A comprehensive discussion can be found for DMTMM in the literature, and analogous considerations also apply to CDMT [4].

## 4. Conclusions

This study demonstrates that the binary CDMT/NMM system represents a highly effective and sustainable zero-length crosslinking strategy for leather tanning. Through triazine-mediated activation of collagen carboxyl groups followed by direct amide bond formation with amino functionalities, the process stabilizes collagen without incorporating external crosslinking structures into the matrix, yielding non-toxic leather with excellent performance.

A key outcome of this work is the mechanistic elucidation of the tanning process. The results show that the in situ generation of reactive triazine intermediates governs the balance between reaction kinetics and diffusion within the collagen network. In fact, rapid addition of the tanning agent leads to heterogeneous crosslinking and wrinkling defects, while controlled, stepwise addition promotes uniform penetration and homogeneous stabilization. These mechanistic insights explain the superior performance of CDMT/NMM compared with DMTMM and provide a rational basis for designing more efficient triazine-based tanning systems.

The optimized process achieved high shrinkage temperatures (81–85 °C) at remarkably low reagent loadings (2.5–3.4 wt%), comparable to glutaraldehyde tanning and superior to many reported metal-free alternatives. The resulting leathers exhibited a bright white color, excellent tear resistance, competitive tensile properties, and suitability for automotive, upholstery, and selected footwear applications. Additionally, the use of CDMT/NMM avoids retention of metals and external toxic crosslinking agents within the collagen matrix, reduces chemical consumption, improves wastewater biodegradability, and contributes to cleaner waste streams. Together with the elimination of pickling and the absence of metals and aldehydes, these features position CDMT/NMM as a promising next-generation tanning technology.

Despite these promising results, certain limitations of the current study must be acknowledged. The evaluation was conducted primarily at laboratory and pilot scales; thus, full industrial-scale validation, process robustness under variable hide conditions, and detailed techno-economic feasibility remain to be established. Furthermore, while initial material properties are highly competitive, the long-term aging behavior, chemical durability, and comprehensive life cycle assessment (LCA) of CDMT/NMM tanned leathers require further investigation. Addressing these limitations defines the roadmap for future research. Ongoing work is focused on optimizing retanning formulations, scaling up the process to industrial drums, and thoroughly evaluating the long-term durability and environmental benefits of this system. Overall, by combining reduced chemical inputs, elimination of hazardous substances, and enhanced process efficiency, the CDMT/NMM system offers a viable, safer, and more sustainable candidate for next-generation leather manufacturing.

## Figures and Tables

**Table 1:** Tanning tests with CDMT/NMM: screening of tanning agent wt%, number of rates, pH variation, and Ts values.

Entry	TA ^1^ (wt%)	n° Rates	T_in_ ^2^ (°C)	pH_in_ ^3^	pH_fin_ ^4^	T_s_ ^5^ (°C)
T0	8.4	1	15.0	7.8	5.8	85.0
T1	8.4	4	15.0	7.8	5.8	85.0
T2	5.9	4	15.0	7.8	5.8	83.0
T3	5.9	4	25.2	7.8	5.8	83.0
T4	5.9	4	33.7	7.8	5.7	78.0
T5	4.4	3	18.5	7.2	5.7	82.5
T6	3.4	3	20.2	7.8	5.5	85.5
T7	2.5	3	20.2	7.8	5.5	83.5
T8	2.5	3	24.5	7.8	5.8	81.0
T9 ^6,7^	2.5	1	25.0	3.0	4.2	80.0
T10 ^8^	2.5	1	20.0	7.0	5.7	73.0

^1^ TA: Tanning agent (WT%). ^2^ T_in_: initial temperature of the water. ^3^ pH_in_: initial pH of the water. ^4^ pH_fin_: pH of the water after tanning. ^5^ T_s_: collagen shrinking temperature after tanning. ^6^ Tanning agent: glutaraldehyde (GTA); ^7^ 3.5 wt% syntan was added 60 min after the addition of glutaraldehyde. ^8^ Tanning agent: DMTMM.

**Table 2:** Physical-mechanical and organoleptic properties of crust leathers retanned with CDMT/NMM and glutaraldehyde, compared with commercial application standards.

Sample	T3-R1	T3-R2	T8-R3	T9-R3	Upholstery	Upper Footwear	Automotive
T_s_ ^1^	83 ± 0.1	83 ± 0.3	81 ± 0.2	80 ± 0.7	75–80	75–80	75–80
TS ^2^	15.9 ± 1.8	13.8 ± 4.8	9.0 ± 0.3	12.3 ± 1.0	≥8	≥15	≥10
E% ^3^	90.0 ± 2.0	73.9 ± 4.0	88.0 ± 20.0	68.5 ± 10.0	35–75	35–60	35–60
TL ^4^	94.6 ± 5.0	135.3 ± 23.0	49.0 ± 1.0	97.2 ± 1.5	≥60	≥100	≥40
MF ^5^	256.3 ± 22.0	208.8 ± 70.0	117.7 ± 2.0	163.4 ± 3.2	≥150	≥200	≥120
DC ^6^	5.6	5.0	5.0	5.5	6–10	6–10	6–10
FC ^7^	446.0	256.0	192.0	323.3	≥180	≥200	≥200
Color ^8^	20 hits 4/5	20 hits 4	100 hits 5	150 hits 4/5	4–5	20 hits ≥ 3	500 hits ≥ 5
HS ^9^			3%	5%			3%

^1^ Ts: Shrinkage temperature in wet conditions calculated from average values parallel and perpendicular tests (Figures S4–S7) (°C), ISO 3380:2015 [55]. ^2^ TS: Tensile strength (MPa), ISO 3376:2020 [56]. ^3^ E: Elongation at break (%), ISO 3376:2020 [56]. ^4^ TL: Tear Load (N), ISO 3377-1:2011 [57]. ^5^ MF: Maximum tensile force at break (N), ISO 3376:2020 [56]. ^6^ DC: distension and strength surface crack (mm) (ball burst method; ISO 3379:2015) [58]. ^7^ FC: Force at crack (N), ISO 3379:2015 [58]. ^8^ Color: Color fastness: Gray Scale evaluation, ISO 11640:2018 [59]. ^9^ HS: Hot shrinkage (%), Fiat specifications 9.55253 [60].

**Table 3:** Characteristics of tanning wastewater: COD, BOD, solids content, dissolved solids, dissolved chlorines, and Cr(III) concentration.

TA ^1^	wt% ^2^	pH ^3^	COD	BOD	BOD_5_/COD	TSC	TDS	TSS	TDC	Cr(III)	Ref.
CTP ^4^	5–10	2.5	2.0–5.0	0.6–0.9	0.18–0.3	30.0–60.0	29.0–58.0	1.0–2.5	15.0–25.0	1.5–4.0	[22,27]
GTA ^5^	5–10	4.5	3.0–7.0	0.3–0.6	0.20–0.25	20.0–40.0	12.0–20.5	0.4–1.0	n.d.	0	[23]
Triazine ^6^	10–15	3.0–6.0	5.0–13.2	0.3–4.6	0.35–0.58	20.0–40.0	8.5–40.7	0.4–1.2	1.2–10.0	0	[16,21,23,25,64]
DMTMM	5–10	7.2–7.8	5.0–8.0	3.2–4.6	0.58–0.64	25.0–35.0	22.2–34.5	0.8–1.2	5.5–6.0	0	[4]
CDMT/NMM	2.5–3.4	7.2–7.8	2.0–5.0	1.8–3.2	0.64–0.90	13.5–21.0	11.3–22.0	0.4–0.9	1.4–2.2	0	This work

^1^ TA: Tanning agent. ^2^ wt%: weight or tanning agent by weight of hide processed. ^3^ pH of the solution before tannage. ^4^ CTP: Chrome-tanning process. ^5^ GTA: Glutaraldehyde. ^6^ Triazine: an average of data available in the literature is reported [4,16].

## Data Availability

The original contributions presented in this study are included in the article/Supplementary Materials. Further inquiries can be directed to the corresponding author.

## Supplementary Materials

The following supporting information can be downloaded at: https://www.mdpi.com/article/10.3390/polym18161968/s1. Table S1. Tanning protocol of test T3, Table S2. Tanning protocol of test T8; Table S3. Tanning protocol of test T9. Table S4. R1 retanning formulation for footwear. Table S5. R2 retanning formulation for automotive and upholstery. Table S6. R3 sustainable retanning formulation for automotive and upholstery. Table S7. Comparison of CDMT/NMM tanning performance with respect to SACC-bases and other triazine systems reported in the literature. Figure S1. ATR FTIR of untanned hide and CDMT + NMM tanned leather. Figure S2. SEM image of untanned hide at 500× magnification. Figure S3. SEM image of CDMT + NMM tanned leather at 500× magnification. Figure S4. Parallel and perpendicular Max force (MF, N) values of samples compared. Figure S5. Parallel and perpendicular Tensile strength (TS, MPa) values of samples compared. Figure S6. Parallel and perpendicular Elongation at break (E%) values of samples compared. Figure S7. Parallel and perpendicular tear load (TL, N) values of samples compared. Figure S8. Test samples. References [4,19,20,21,22,23,25] are cited in the Supplementary Materials.

## Abbreviations

The following abbreviations are used in this manuscript:

BOD	Biochemical oxygen demand
CDMT	2-chloro-4,6-dimethoxy-1,3,5-triazine
COD	Chemical oxygen demand
CTP	Chrome-tanning process
DMTMM	4-(4,6-dimethoxy-1,3,5-triazin-2-yl)-4-methylmorpholinium chloride
DC	Distension and strength surface at crack
DS	Distention at crack
E%	Elongation at break
FC	Force at crack
GTA	Glutaraldehyde
HS	Hot shrinkage
MF	Max force
NMM	*N*-methylmorpholine
SACC	Sodium *p*-((4,6-dichloro-1,3,5-triazin-2-yl)amino)benzene sulphonate
TDC	Total dissolved chlorides
TL	Tear load
Ts	Shrinkage temperature
TS	Tensile strength
TSD	Total dissolved solids
TSC	Total solid content
TSS	Total suspended solids
