# Supplementary material for: Triazine-Mediated Zero-Length Crosslinking for Sustainable Leather Tanning
Source: Polymers (Basel). 2026 Aug 12;18(16):1968. doi: 10.3390/polym18161968 (PMC13516574; doi:10.3390/polym18161968)
Supplement: Supplementary file 1 [file polymers-18-01968-s001.zip › polymers-4473604-supplementary.pdf]

Supplementary Material

Triazine-Mediated Zero-Length Crosslinking for Sustainable Leather Tanning

Valentina Beghetto[1,2,3]*, Eleonora Fabris[1], Francesco de Laurentiis[4], Marco Nogarole[4], Domenico Santandrea[1], Dior Tall[1]

[1] Department of Molecular Sciences and Nanosystems, University Ca' Foscari of Venice, Via Torino 155, 30172 Mestre, Italy.

[2] Crossing S.r.l., Viale della Repubblica 193/b, 31100 Treviso, Italy.

[3] Consorzio Interuniversitario per le Reattività Chimiche e La Catalisi (CIRCC), Via C. Ulpiani 27, 70126, Bari, Italy

[4] Stazione Sperimentale Per L'industria Delle Pelli E Delle Materie Concianti Srl, Via del Lavoro, 22, 36071 Arzignano (VI) – Italia.

*Corresponding Author: beghetto@unive.it;

Index

Table S1. Tanning protocol of test T3 pag. 2

Table S2. Tanning protocol of test T8 pag. 2

Table S3. Tanning protocol of test T9 pag. 3

Table S4. R1 retanning formulation for footwear pag. 3

Table S5. R2 retanning formulation for automotive and upholstery pag. 4

Table S6. R3 sustainable retanning formulation for automotive and upholstery pag. 5

Table S7. Comparison of CDMT/NMM tanning performance with respect to SACC-bases and other triazine systems reported in the literature pag. 5

Figure S1. ATR FTIR of untanned hide and CDMT+NMM tanned leather. pag. 6

Figure S2. SEM image of untanned hide at 500x magnification pag. 6

Figure S3. SEM image of CDMT+NMM tanned leather at 500x magnification pag. 7

Figure S4. Parallel and perpendicular Max force (MF, N) values of samples compared pag. 7

Figure S5. Parallel and perpendicular Tensile strength (TS, MPa) values of samples compared pag. 8

Figure S6. Parallel and perpendicular Elongation at break (E%) values of samples compared pag. 8

Figure S7. Parallel and perpendicular tear load (TL, N) values of samples compared pag. 8

Figure S8. a) Leather direction: parallel and perpendicular to the backbone; b) sample used for tensile strength and percentage elongation test; c) sample used for tear load test; d) sample used for surface tensile strength test; e) felt pads used for colour fastness to cycles of to-and-fro rubbing test. pag. 9

Table S1. Tanning protocol of test T3.

The test was carried out on a sample of bull hide with a raw weight of 3.000 kg.

Rates	CDMT wt%	NMM wt%	Water wt%	Time after additions	pH		T		T_s
					before addition	after addition	before addition	after addition	
1	0.88	0.60	7.5	2 h	7.8	5.7	33.7	27.9	65
2	0.88	0.60	7.5	2 h	5.7	5.7	27.9	24.6	69
3	0.88	0.60	7.5	2 h	5.7	5.7	24.6	25.6	78
4	0.88	0.60	7.5	18 h	5.7	5.7	25.6	25.3	78

Table S2. Tanning protocol of test T8.

The test was carried out on a sample of bull hide with a raw weight of 9.6 kg.

Rates	CDMT wt%	NMM wt%	Water wt%	Time after additions	pH		T		T_s
					before addition	after addition	before addition	after addition	
1	0.67	0.47	10	2 h	7.8	6.6	24.5	25	63
2	0.67	0.47	10	2 h	6.6	6.3	25	27.6	69
3	0.67	0.47	10	18 h	6.3	5.8	27.6	23.6	81

Table S3. Tanning protocol of test T9.

The test was carried out on a sample of bull hide with a raw weight of 7.8 kg.

Product	Quantity (wt %)	Dilution	Temperature (°C)	Time (min)	pH
Water	50	-	-	-	-
Glutaraldehyde (25 wt%)	2.5	1:3	25	60	3
4,4′-sulfonyldiphenol	3.5	-	25	120	-
Sodium Formiate	1.0	-	25	30	4
Sodium Bicarbonate	0.5	1:10	25	-	4.2

Table S4. R1 retanning formulation for footwear.

Product	Quantity (wt %)	Dilution	Temperature (°C)	Time (min)
Water	100	-	20	-
Auxiliary tannin naphthalenesulfonic-based	2	-		5
Replacement tannin phenolic-based	6	-		5
Sulphonated oil	6	1:3	50	60
Drain				
Water	100	-	20	-
Replacement tannin phenolic-based	10	-		
Dicyandiamide-based resin	3	-		60
Colorant: Acid black 2, anionic	3	-	50	30
Sulphonated oil	4	1:3	50	40

Replacement tannin phenolic-based	4	-	50	40
Tara powder	4	-	50	20
Methacrylic resin	2	-	50	30
Formic Acid	3.5	1:5	50	20

Table S5. R2 retanning formulation for automotive and upholstery.

Product	Quantity (wt %)	Dilution	Temperature (°C)	Time (min)
Water	100	-	20	-
Sulphonated fish oil	8	1:3		
Sulphosuccinate	10	1:3	50	60
Melamine-based resin	3	-		
Substitution phenolic syntan	3	-	20	60
Formic Acid	1	1:5	50	15
Water	100	-	20	-
Auxiliary tannin naphthalenesulfonic-based	2	-		
Colorant: Acid brown 282, premetallised	3	-		60
Formic Acid	3	1:5	50	30

Table S6. R3 sustainable retanning formulation for automotive and upholstery.

Product	Quantity (wt %)	Dilution	Temperature (°C)	Time (min)
Water	100	-	20	-
Sulphonated fish oil	10	1:3		
Sulphosuccinate	10	1:3	50	60
Methacrylic resin	4	-		
Tara powder	5	-		60
Formic Acid	1	1:10	20	15
Water	100	-	20	-
Colorant: Acid brown 282	3	-		60
Water	100	-		-
Formic Acid	2	1:10	50	30

Table S7. Comparison of CDMT/NMM tanning performance with respect to SACC-bases and other triazine systems reported in the literature.

Tanning systems	Reagent loading (wt% of hide)	Pickling	Ts(°C)	Auxiliary tanning agent	Ref.
SACC (Granofin F90®	4	No	~78	No	[19]
SACC + Tannic Acid	4-6	No	78-82	Yes	[21,22]
Other triazine systems	5-10	No	75-82	Yes	[20,23,25]
DMTMM	5	No	76	No	[4]
CDMT/NMM	2.5-3.4	No	81-85	No	This work

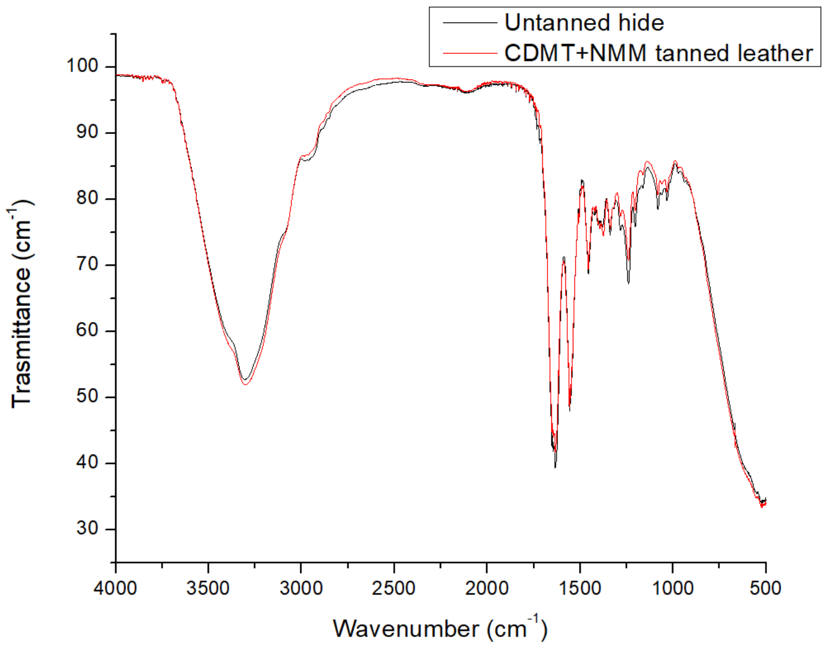

Figure S1. ATR FTIR of untanned hide and CDMT+NMM tanned leather

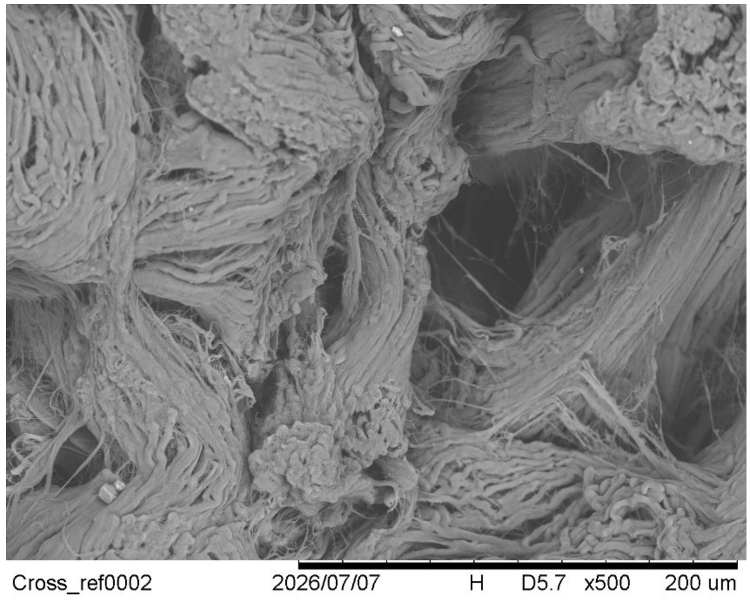

Figure S2. SEM image of untanned hide at 500x magnification

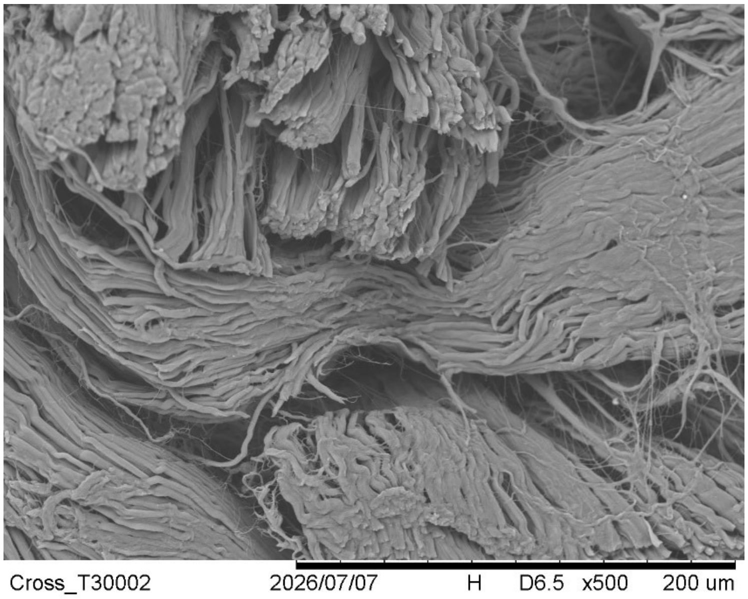

Figure S3. SEM image of CDMT+NMM tanned leather at 500x magnification

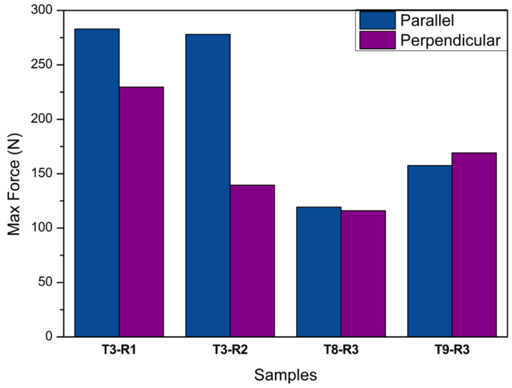

Figure S4. Parallel and perpendicular Max force (MF, N) values of samples compared

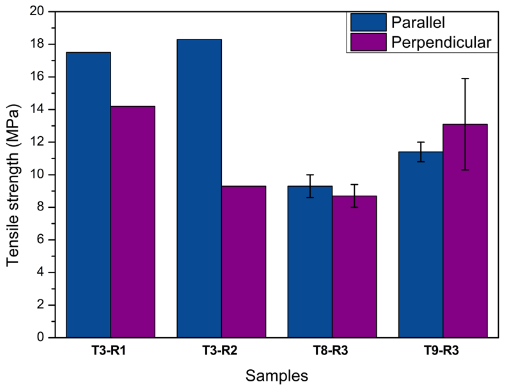

Figure S5. Parallel and perpendicular Tensile strength (TS, MPa) values of samples compared

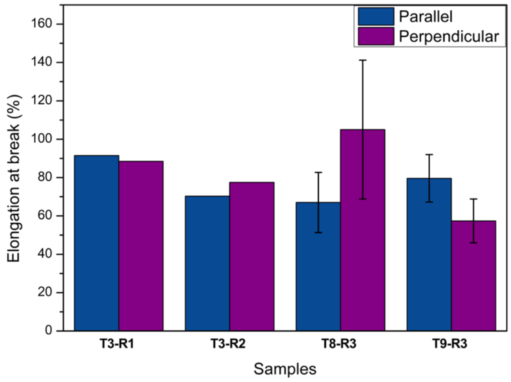

Figure S6. Parallel and perpendicular Elongation at break (E%) values of samples compared

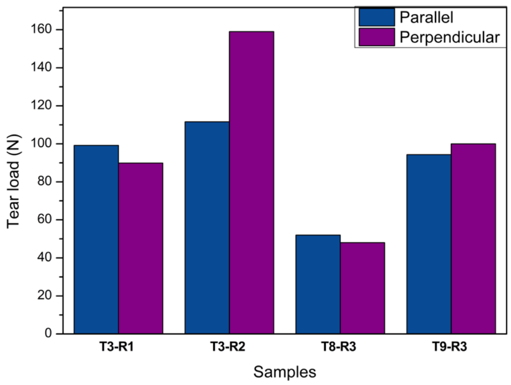

Figure S7. Parallel and perpendicular tear load (TL, N) values of samples compared

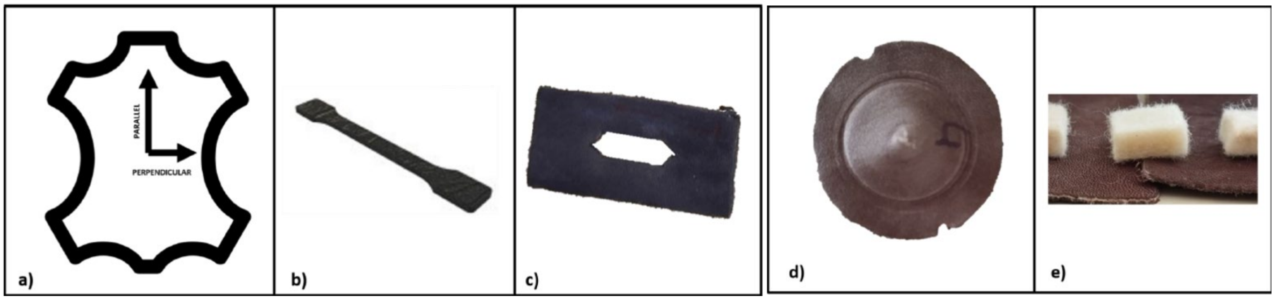

Figure S8. a) Leather direction: parallel and perpendicular to the backbone; b) sample used for tensile strength and percentage elongation test; c) sample used for tear load test; d) sample used for surface tensile strength test; e) felt pads used for colour fastness to cycles of to-and-fro rubbing test.
